# Automated diagnosis and grading of lumbar intervertebral disc degeneration based on a modified YOLO framework

**DOI:** 10.3389/fbioe.2025.1526478

**Published:** 2025-01-22

**Authors:** Aobo Wang, Tianyi Wang, Xingyu Liu, Ning Fan, Shuo Yuan, Peng Du, Congying Zou, Ruiyuan Chen, Yu Xi, Zhao Gu, Hongxing Song, Qi Fei, Yiling Zhang, Lei Zang

**Affiliations:** ^1^ Department of Orthopedics, Beijing Chaoyang Hospital, Capital Medical University, Beijing, China; ^2^ School of Life Sciences, Tsinghua University, Beijing, China; ^3^ Department of Biomedical Engineering, School of Medicine, Tsinghua University, Beijing, China; ^4^ Institute of Biomedical and Health Engineering (iBHE), Tsinghua Shenzhen International Graduate School, Shenzhen, China; ^5^ Longwood Valley Medical Technology Co. Ltd, Beijing, China; ^6^ Department of Orthopedics, Beijing Shijitan Hospital, Capital Medical University, Beijing, China; ^7^ Department of Orthopedics, Beijing Friendship Hospital, Capital Medical University, Beijing, China

**Keywords:** deep learning, diagnosis, magnetic resonance imaging, artificial intelligence, intervertebral disc degeneration

## Abstract

**Background:**

The high prevalence of low back pain has led to an increasing demand for the analysis of lumbar magnetic resonance (MR) images. This study aimed to develop and evaluate a deep-learning-assisted automated system for diagnosing and grading lumbar intervertebral disc degeneration based on lumbar T2-weighted sagittal and axial MR images.

**Methods:**

This study included a total of 472 patients who underwent lumbar MR scans between January 2021 and November 2023, with 420 in the internal dataset and 52 in the external dataset. The MR images were evaluated and labeled by experts according to current guidelines, and the results were considered the ground truth. The annotations included the Pfirrmann grading of disc degeneration, disc herniation, and high-intensity zones (HIZ). The automated diagnostic model was based on the YOLOv5 network, modified by adding an attention module in the Cross Stage Partial part and a residual module in the Spatial Pyramid Pooling-Fast part. The model’s diagnostic performance was evaluated by calculating the precision, recall, F1 score, and area under the receiver operating characteristic curve.

**Results:**

In the internal test set, the model achieved precisions of 0.78–0.91, 0.90–0.92, and 0.82 and recalls of 0.86–0.91, 0.90–0.93, and 0.81–0.88 for disc degeneration grading, disc herniation diagnosis, and HIZ detection, respectively. In the external test set, the precision values for disc degeneration grading, herniation diagnosis, and HIZ detection were 0.73–0.87, 0.86–0.92, and 0.74–0.84 and recalls were 0.79–0.87, 0.88–0.91, and 0.77–0.78, respectively.

**Conclusion:**

The proposed model demonstrated a relatively high diagnostic and classification performance and exhibited considerable consistency with expert evaluation.

## 1 Introduction

Approximately 70%–85% of people worldwide experience symptoms of lower back pain (LBP) and leg pain at least once in their lives (de Souza et al., 2019). Such discomfort can significantly affect individuals’ quality of life and increase the healthcare burden. The etiologies of LBP and leg pain are highly complex, with degenerative changes in the lumbar spine being the most common factors (Knezevic et al., 2021). From a pathophysiological perspective, intervertebral disc pathology is closely linked to overall degenerative changes in the spine, leading to related symptoms (Dowdell et al., 2017). First, deformities such as disc herniation can directly compress the dural sac or nerve roots. Second, annulus fibrosus rupture of the intervertebral disc can result in the release of pain factors and inflammatory mediators, stimulating nearby nerve roots. Third, the intervertebral disc has been considered the initiator of overall lumbar degeneration (Leone et al., 2007). Briefly, disc collapse and weakened stress can reduce lumbar stability, influence stress distribution, and further accelerate degenerative changes in other structures such as the facet joint and ligament flavum.

For symptomatic patients, magnetic resonance imaging (MRI) of the lumbar spine is a commonly used diagnostic tool in outpatient settings. Given the crucial role of the intervertebral disc, it is a primary focus in the radiological assessment of the lumbar spine (Kamei et al., 2022). MRI can clearly depict the degree of disc degeneration and disc herniation. This information aids doctors in making accurate diagnoses and developing treatment plans, such as guiding decompression operations. However, the interpretation of lumbar MRI remains a complex and subjective process, heavily dependent on the clinical experience of the clinician (Liawrungrueang et al., 2024). Moreover, current clinical resources are under increasing strain due to the rising demand from patients (Chen et al., 2022).

With the rapid development of artificial intelligence (AI) technology, computer-aided diagnosis can improve clinical workflows (Kulkarni and Singh, 2023; D'Antoni et al., 2021). A decade ago, machine learning was already being explored for diagnosing intervertebral disc degeneration using small-scale MRI datasets (Alomari et al., 2010; Koh, Chaudhary, and Dhillon, 2012). In recent years, deep learning (DL), a state-of-the-art AI approach, leverages specialized neural network architectures to process raw data, automatically extracting hierarchical and abstract features through multi-layered, nonlinear networks (LeCun, Bengio, and Hinton, 2015; Huang et al., 2024). These capabilities enable accurate and efficient image detection and classification. DL has shown remarkable potential in the imaging evaluation of intervertebral discs. Achieving good performance in grading disc degeneration and detecting disc herniation (Zheng et al., 2022; Tsai et al., 2021; Nikpasand et al., 2024; Soydan et al., 2023). Such advancements may enhance clinical efficiency, aid in identifying overlooked lesions, and improve diagnostic accuracy, particularly for junior and primary care clinicians (Compte et al., 2023; Martin-Noguerol et al., 2023).

However, there are still gaps remain that limit the clinical application of deep learning technologies. First, previous studies on the assessment of intervertebral disc degeneration have been narrow. In addition to disc herniation, other radiological manifestations of degeneration, such as high-intensity zones (HIZ), are also considered common causes of LBP (Wang and Hu, 2012). To our knowledge, no prior studies have explored automatic detection methods for this feature. Second, while existing models for diagnosing and grading disc degeneration have demonstrated promising performance, they are typically designed with complex and multi-stage algorithms that separately handle image segmentation, detection, and classification. Such models, while effective in research context, face practical limitations in clinical settings where simpler and more streamlined solutions are needed (He et al., 2024). By avoiding the use of overly complex algorithms, these models can improve their clinical applicability and facilitate broader implementation in routine practice.

In this study, a multitask automatic diagnostic model for lumbar intervertebral disc degeneration was developed based on a modified YOLOv5 network. The key advantage of this model lies in its ability to simultaneously handle multiple detection and classification tasks. Utilizing clinical MR images, this model can qualitatively assess the degree of lumbar intervertebral disc degeneration, including the evaluation of Pfirrmann grades of disc degeneration, disc herniation diagnosis, and HIZ identification. Drawing on the experience from the widely adopted chest CT auxiliary diagnostic systems (Xudong et al., 2022), the proposed model primarily aims to provide simple and clear outputs—the imaging slice and the location of specific lesions. Such systems have demonstrated their effectiveness in supporting clinicians with image interpretation and report generation.

## 2 Materials and methods

### 2.1 General guidelines

This study was conducted and reported in accordance with the Standards for Reporting of Diagnostic Accuracy criteria (see Supplementary Material). This study was conducted in accordance with the Declaration of Helsinki. The study was approved by the institutional ethics committee (2024-KE-385). Since retrospective studies do not involve any additional intervention and privacy disclosure, the informed consent requirement was waived.

### 2.2 Data collection

**FIGURE 1:**
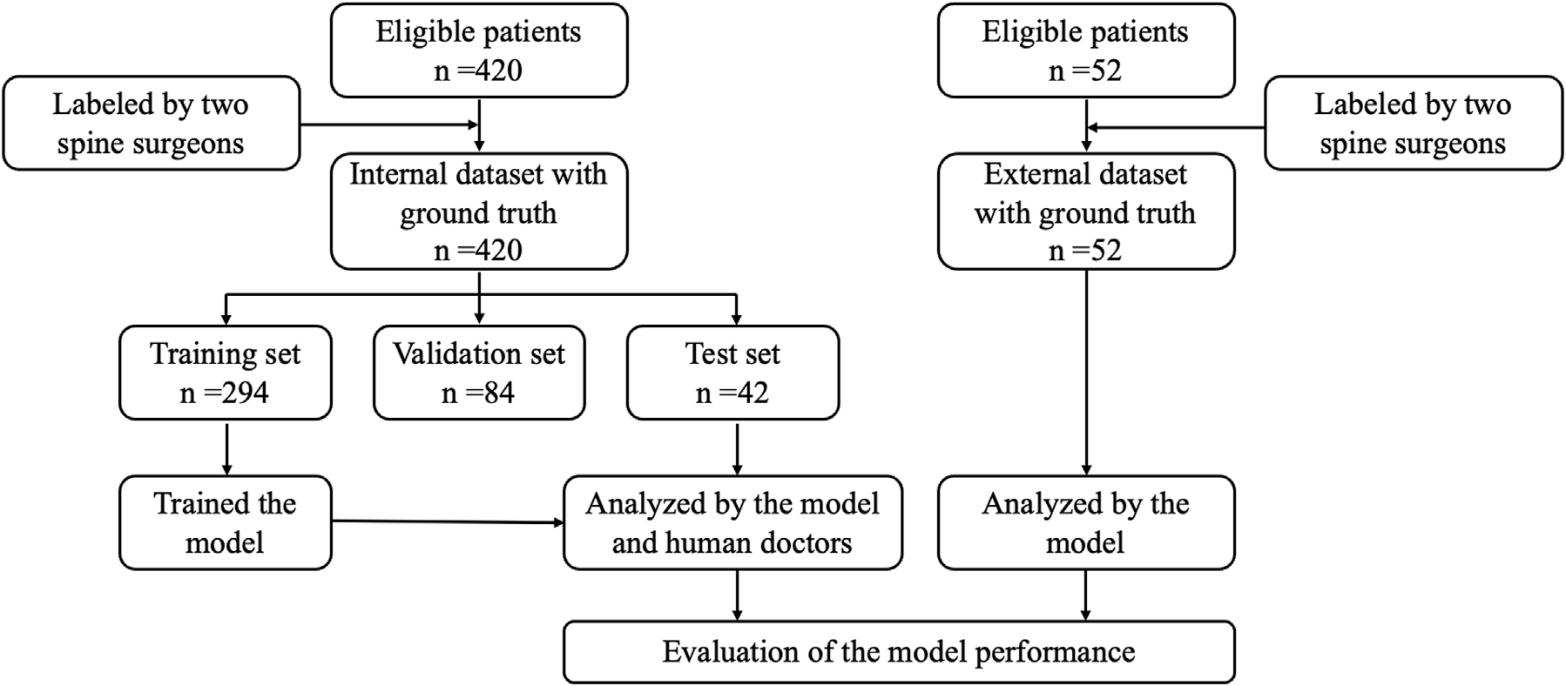
Data allocation and processing flowchart.

Figure 1 depicts the data allocation and processing workflow. From January 2021 to November 2023, a dataset of 10,028 axial and 5,040 sagittal T2-weighted (T2W) MR images was collected from 420 patients with LBP or leg pain at Beijing Chaoyang Hospital, Capital Medical University. In addition, an external testing set included a total of 1,228 axial and 624 sagittal T2W MR images from 52 patients at Beijing Shijitan Hospital, Capital Medical University, and Beijing Friendship Hospital, Capital Medical University. All data were anonymized and numbered.

Patients aged >18 years undergoing lumbar spine MRI at an outpatient or inpatient clinic were included. Individuals with spinal infection, trauma, or deformity; history of malignant tumors; history of previous spinal surgery, particularly with implants; and poor image quality, such as image blurring caused by patient movement or the presence of artifacts affecting imaging evaluation, were excluded.

Considering the patient population in the hospital, the proportion of certain degeneration patterns is relatively low, such as disc degeneration grades 1 and 5, as well as HIZ (see the “Dataset Labeling and Reference Standard” section). Therefore, the 420 patients included in our study do not constitute a consecutive cohort. This group consisted of 246 consecutive patients, supplemented by additional 124 patients with HIZ, 20 patients aged <30 years, and 30 patients aged >75 years. Regarding the distribution of chief complaints, 133 patients presented with LBP, while 287 patients reported leg pain or claudication.

In the internal dataset, MRI scans were performed using a 3.0-T MRI scanner (Magnetom Verio, Siemens Healthcare, Erlangen, Germany). In the external dataset, MRI scans were performed using the Discovery MR 750 W 3.0TMR scanner (GE Medical Systems, Milwaukee, WI, United States). The core parameters set for the MRI systems are provided in Table 1.

**TABLE 1:** MRI scanning parameters for T2-weighted axial and sagittal scans.

	Internal dataset	External dataset
TR/TE	3,000–3,100/95–110 ms	2,500/128 ms
Axial matrix	512 × 512	512 × 512
Axial slice thickness	3 mm	3 mm
Axial spacing between slices	3.3 mm	3.3 mm
Sagittal matrix	320 × 320	320 × 240
Sagittal slice thickness	4 mm	4 mm
Sagittal spacing between slices	4.8 mm	4.8 mm

### 2.3 Dataset labeling and reference standard

Two spine surgeons with >10 years of experience independently evaluated and annotated each image. In cases where their assessments differed, a third senior surgeon adjudicated the disagreement. In addition, after the annotations were completed, the author compared the annotations with existing radiologist reports and excluded images with significant discrepancies. Thus, the ground truth was established.

**FIGURE 2:**
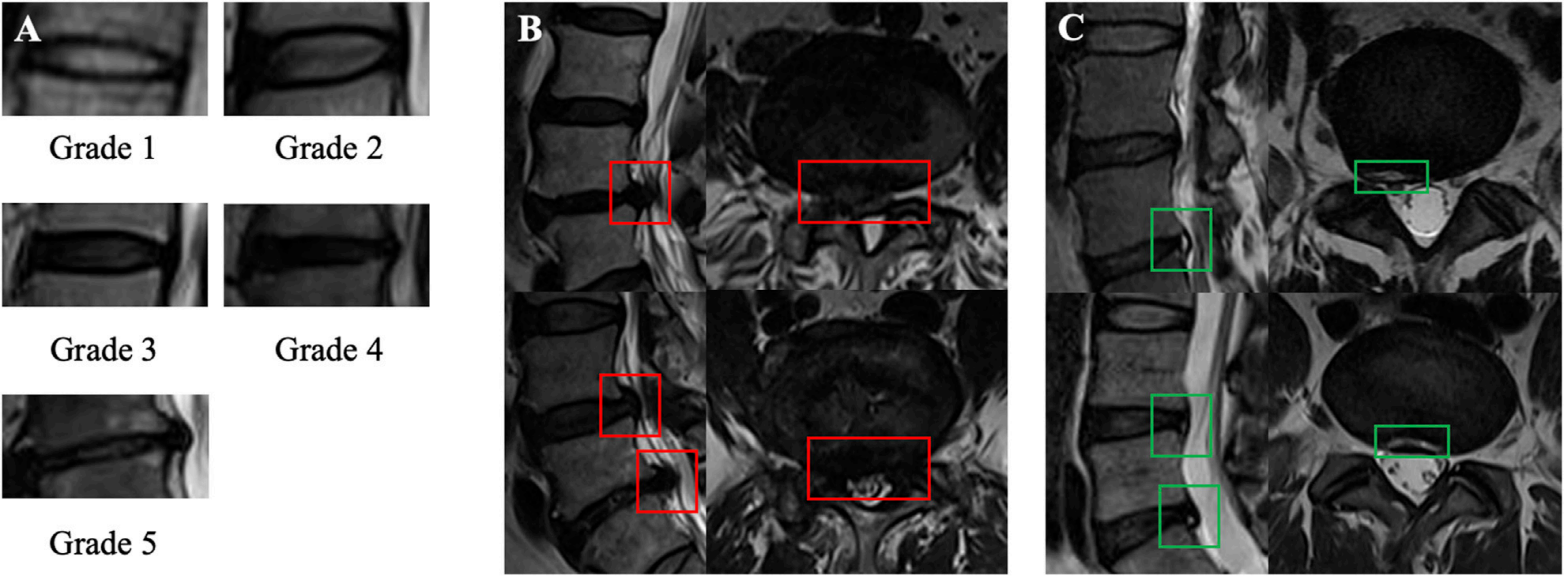
Illustration of the lesions of interest and labeling process. **(A)** Pffirmann grading system (grades 1–5). **(B)** Lumbar disc herniation on sagittal and axial MRI. **(C)** High-intensity zone on sagittal and axial MRI.

The diagnostic annotation reference standards used were as follows (Figure 2): disc degeneration was graded according to the Pfirrmann classification system (Pfirrmann et al., 2001). This can be briefly described in five grades: Grade 1 represents a homogeneous bright white nucleus pulposus with a clear boundary from the annulus fibrosus and normal disc height. Grade 2 represents an inhomogeneous nucleus pulposus, with or without horizontal bands, but still relatively clear boundaries. Grade 3 indicates a nucleus pulposus with decreased signal intensity, unclear boundaries, and possibly mild disc height reduction. Grade 4 represents a heterogeneous gray or black nucleus pulposus, where the nucleus pulposus and annulus fibrosus are indistinguishable. Grade 5 indicates that the entire disc shows low-signal intensity with disc space collapse. According to the commonly used Jensen grading system (Li, Fredrickson, and Resnick, 2015), lumbar disc protrusion or extrusion, defined as a partial or extensive extension beyond the interspace, was considered disc herniation. Mild disc bulging was not considered herniation. HIZ was defined as a high-intensity signal within the low-signal annulus fibrosus on T2W MR images (Teraguchi et al., 2020). HIZ can be located anteriorly or posteriorly in the disc but is clearly dissociated from the nucleus pulposus and shows higher signal intensity than the nucleus pulposus.

All annotations were completed using the Labelme program. Although Pfirrmann’s description states that disc degeneration grading should be based on the mid-sagittal slice, all visible discs in the mid-sagittal slice and two para-midsagittal slices were annotated to enhance data volume and clinical applicability, as all these slices provide sufficient disc information. For disc herniation and HIZ, a positive annotation strategy was used, that is, marking diagnostic positive results in all sagittal and axial images, without separately annotating normal discs.

### 2.4 Data preprocessing

The internal dataset was randomly divided into training (n = 294), validation (n = 84), and testing (n = 42) sets. To improve the model’s generalization and performance on test data and further applications, data augmentation was performed on the training set. The specific methods included: adding Gaussian noise to the images to simulate real-world noise interference and increase data diversity and applying gamma transformation, which is a nonlinear operation used for image enhancement to adjust the contrast and brightness of the images. A gamma transformation with random values ranging from 0.8 to 1.2 was applied. 30% of the original data were randomly selected and transformed using the two methods.

### 2.5 Construction of the deep-learning network model

**FIGURE 3:**
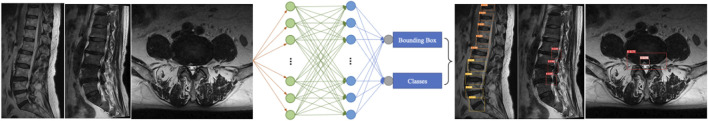
Overview of the deep learning pipeline. The modified YOLOv5 model is capable of simultaneously detecting and classifying three types of lesions based on sagittal and axial lumbar MRI.

**FIGURE 4:**
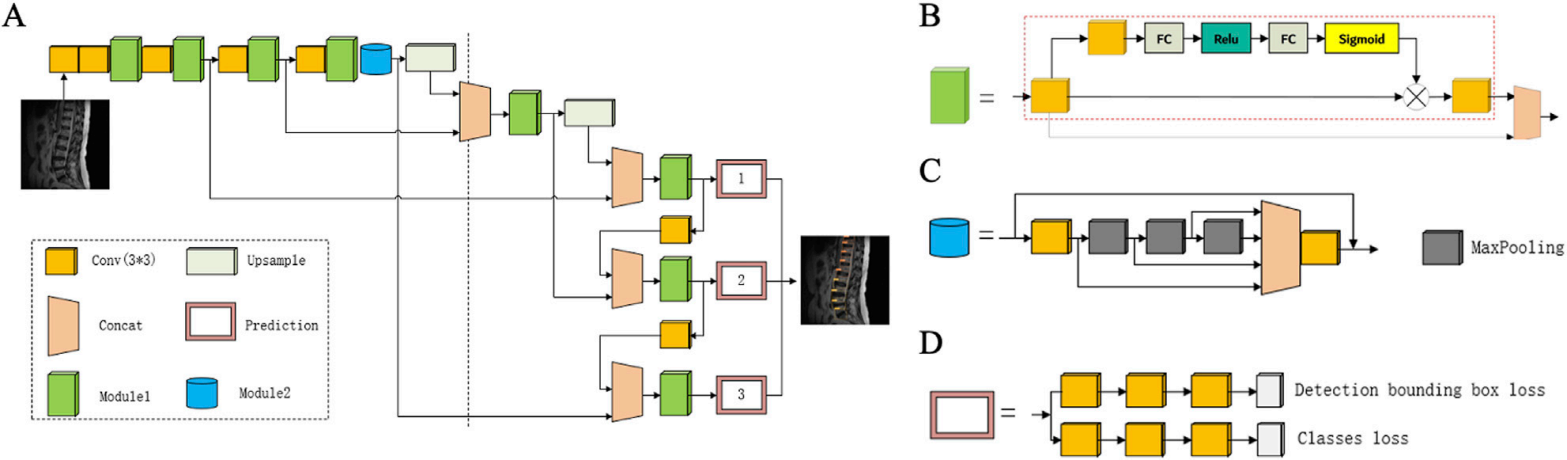
Design of the proposed deep learning network. **(A)** Overview of the proposed network architecture; **(B)** Cross Stage Partial module integrated with an SE-like attention mechanism; **(C)** Spatial Pyramid Pooling-Fast module based on residual design; **(D)** Schematic representation of the prediction module.

Figures 3, 4 illustrates the proposed deep-learning network model. The study utilized a modified YOLOv5 network as the algorithm framework. In the Neck network, YOLOv5 uses a feature pyramid network (FPN), which can perform detection at different feature map levels to enhance object detection performance by integrating information from various feature layers. The head network consists of three output layers, each of which is responsible for detecting large, medium, and small-scale objects. To ensure the accuracy of small object detection, the network was improved by adding an attention module in the Cross Stage Partial (CSP) part (Module 1) and a residual module in the Spatial Pyramid Pooling-Fast (SSPF) part (Module 2).

#### 2.5.1 Module 1

The improvement in the CSP module is shown in Module 1. This module employs a squeeze-and-excitation (SE)-like attention mechanism to extract dependencies between channels. The SE-like attention mechanism mainly consists of Conv3x3 + fully connected (FC) + rectified linear unit (ReLU) + FC + Sigmoid, as indicated by the red dashed box. This module introduces more nonlinearity, allowing for better fitting of complex inter-channel relationships through FC processing while significantly reducing the number of parameters and computational load. The features extracted by the attention mechanism are concatenated with the original feature map to complete the feature fusion.

#### 2.5.2 Module 2

The improvement in the SSPF module is shown in Module 2. This part continuously applies maximum pooling three times and integrates the results of maximum pooling. Given that MR image clarity is not uniform owing to equipment and operator experience, the concept of residuals was introduced. By adding residual calculations on the original basis, the detailed features can be better preserved, thereby enhancing the detection capability for small objects.

#### 2.5.3 Loss function

The classification and bounding box loss functions were employed to address the objectives of detection and classification in this study. The classification loss function was defined as:

$${Cls}_{Loss}=-\sum_{i=1}^{n}\left[y_{i}\times \log\left(y_{i}\prime \right)+\left(1-y_{i}\right)\times \log\left(1-y_{i}\prime \right)\right]$$

Among them, i represents the i -th category, *n* denotes the total number of categories, y is the ground truth label for the i *-th* category, and $y^{\prime }$ is the predicted label for the i -th category.

The bounding box loss was defined as:

$${DIoU}_{Loss}=1-IoU+\frac{\rho^{2}\left(b,b^{gt}\right)}{c^{2}}$$

$$IoU=\frac{box\cap box\prime }{box\cup box\prime }$$

Among them, ρ represents the Euclidean distance between the two rectangular boxes; b denotes the center point of the predicted bounding box; $b^{gt}$ refers to the center point of the ground truth bounding box; box represents the ground truth bounding box, and $box^{\prime }$ refers to the predicted bounding box.

The overall loss function is computed as the average of the classification loss and bounding box loss, ensuring a balanced optimization of both tasks:

$$Loss=\frac{{Cls}_{Loss}+{DIoU}_{Loss}}{2}$$

### 2.6 Statistical analysis

The performance of the automated diagnostic model was evaluated on the internal and external test datasets. For the automated grading of disc degeneration, a confusion matrix of multigrade classification was constructed. Then, quantitative evaluation metrics, including precision, recall, and F1 score, were computed based on the confusion matrices. The evaluation metrics were defined as

$$Precision=\frac{TP}{TP+FP}$$

$$Recall=\frac{TP}{TP+FN}$$

$$F1\;score=\frac{2\times Precision\times Recall}{Precision+Recall}$$

Among them, TP, TN, FP, and FN refer to the number of true positives, true negatives, false positives, and false negatives, respectively. The area under the receiver operating characteristic curve (AUC) was also calculated. For the automated detection of lumbar disc herniation and HIZ, the precision, recall, and F1 score were calculated. The linearly weighted Cohen’s kappa coefficient was calculated to evaluate the classification performance of the deep-learning model and human doctors; p < 0.05 was considered statistically significant.

Considering that the above diagnostic and grading criteria are qualitative rather than quantitative, the diagnosis of the degree of disc degeneration may be ambiguous in clinical practice. Therefore, the slice-wise accuracy of the automated diagnosis was further assessed to evaluate its performance. An independent senior surgeon directly reviewed the output results of the internal test set. The automatic diagnosis result of a scan slice was considered clinically acceptable if the grading error for all intervertebral discs was ≤1 and there were no significant missed diagnoses or misdiagnoses. Furthermore, considering that patients’ diagnostic needs for disc herniation and HIZ are typically disc-wise rather than slice-wise in clinical practice, the disc-wise diagnostic performance of the model was evaluated. Specifically, all slices of a given disc segment were aggregated, and the disc was classified as pathological if one or more lesions were detected.

To further compare the diagnostic and grading capabilities of the proposed deep learning model with those of human doctors, a medical postgraduate student (reader 1) and an attending clinician (reader 2) were invited to independently annotate all images in the internal test set. The performances of (1) the deep learning model, (2) reader 1, and (3) reader 2 were then evaluated by comparing their results with the ground truth.

## 3 Results

### 3.1 Patient information

Table 2 present the general information and intervertebral disc lesion details of the included patients, respectively. The internal dataset consists of 420 patients aged 59.5 ± 13.3 years (range 18–88). The external dataset contained 52 patients aged 55.1 ± 12.1 years (range 23–78). The specific numbers of lesion annotations are provided in Table 3.

**TABLE 2:** Demographic information of included patients.

Characters	Training set (n = 294)	Validation set (n = 84)	Test set (n = 42)	External test set (n = 52)
Age (years)	60.3 ± 13.2	57.6 ± 13.5	58.2 ± 14.1	55.1 ± 12.1
Gender (M/F)	142/152	41/43	22/20	24/28
BMI (kg/m^2^)	24.6 ± 3.5	24.9 ± 3.3	25.0 ± 3.7	24.5 ± 3.0

**TABLE 3:** Number of lesion annotations in the internal and external dataset.

	Internal dataset	External dataset
Pfirrmann grading of disc degeneration		
1	750	82
2	1,634	120
3	2,178	195
4	2,441	175
5	590	52
Disc herniation (axial)^a^	1844	175
Disc herniation (sagittal)	3,632	363
High-intensity zone (axial)^a^	487	44
High-intensity zone (sagittal)	762	65

^a^ The number of slices where the same lesion can be observed may differ between sagittal and axial scans.

### 3.2 Model performance in the internal test set

**FIGURE 5:**
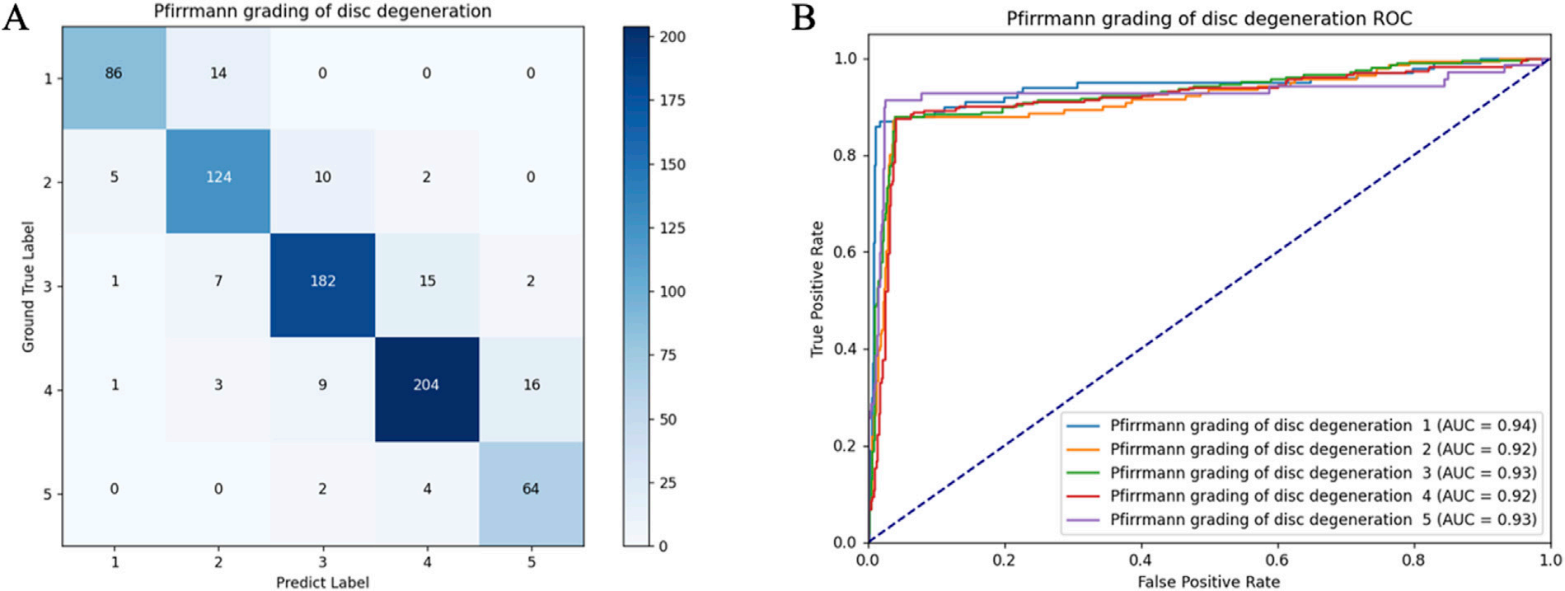
Confusion matrix **(A)** and receiver operating characteristic curves **(B)** of the lumbar disc degeneration automated classification in the internal test set.

Table 4 provides the precision, recall, and F1 score in the internal test set. Generally, the precision values for automated disc degeneration grading, diagnosis of lumbar disc herniation, and HIZ were 0.78–0.91, 0.90–0.92, and 0.82, and recall values of 0.86–0.91, 0.90–0.93, and 0.81–0.88, respectively. Figure 5 shows the confusion matrix and the receiver operating characteristics curve of the disc degeneration automated grading. The disc-wise precision values for the diagnosis of lumbar disc herniation and HIZ was 0.84 and 0.79, respectively, based on sagittal scans, and 0.85 and 0.84, respectively, based on axial scans. The disc-wise recall values for the diagnosis of lumbar disc herniation and HIZ was 0.94 and 0.86, respectively, based on sagittal scans, and 0.94 and 0.89, respectively, based on axial scans.

**TABLE 4:** Diagnostic and classification performance in the internal test set.

	Precision	Recall	F1 score
Disc degeneration grade 1	0.92	0.86	0.89
Disc degeneration grade 2	0.84	0.88	0.86
Disc degeneration grade 3	0.90	0.88	0.89
Disc degeneration grade 4	0.91	0.88	0.89
Disc degeneration grade 5	0.78	0.91	0.84
Lumbar disc herniation (sagittal)	0.92	0.90	0.91
Lumbar disc herniation (axial)	0.90	0.93	0.91
High-intensity zone (sagittal)	0.82	0.88	0.85
High-intensity zone (axial)	0.82	0.81	0.81

Results of the internal testing were independently evaluated by a senior surgeon according to the abovementioned criteria. In this study, 96.8% of the sagittal images and 93.0% of the axial images were considered clinically acceptable. For further comparison of the diagnostic and grading capability of the model and human doctors, Cohen’s kappa coefficients were compared between their results and the ground truth. The coefficients of the model, reader 1, and reader 2 were 0.84, 0.79, and 0.85, respectively.

### 3.3 Model performance in the external test set

**FIGURE 6:**
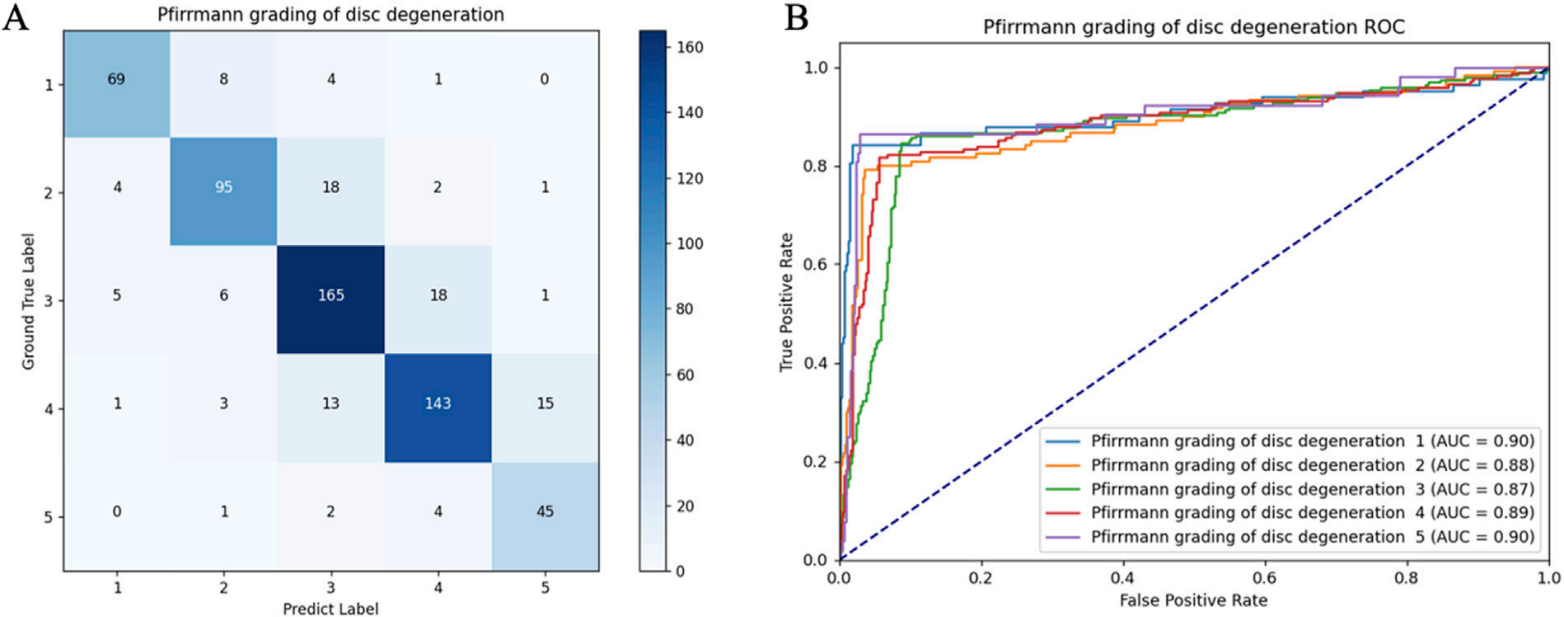
Confusion matrix **(A)** and receiver operating characteristic curves **(B)** of the lumbar disc degeneration automated classification in the external test set.

Table 5 and Figure 6 show the diagnostic and classification performance in the external test set. The precision values of the five disc degeneration grades were 0.87, 0.84, 0.82, 0.85, and 0.73 in sequence. The recall values were 0.84, 0.79, 0.85, 0.82, and 0.87 in sequence. In addition, the precision of disc herniation and HIZ detection were 0.86 and 0.84 in the sagittal images and 0.92 and 0.74 in the axial images. The recalls of disc herniation and HIZ detection were 0.88 and 0.78 in the sagittal images and 0.91 and 0.77 in the axial images. The disc-wise precision values for the diagnosis of lumbar disc herniation and HIZ was 0.79 and 0.78, respectively, based on sagittal scans, and 0.87 and 0.70, respectively, based on axial scans. The disc-wise recall values for the diagnosis of lumbar disc herniation and HIZ was 0.92 and 0.78, respectively, based on sagittal scans, and 0.91 and 0.83, respectively, based on axial scans.

**TABLE 5:** Diagnostic and classification performance in the external test set.

	Precision	Recall	F1 score
Disc degeneration grade 1	0.87	0.84	0.86
Disc degeneration grade 2	0.84	0.79	0.82
Disc degeneration grade 3	0.82	0.85	0.83
Disc degeneration grade 4	0.85	0.82	0.83
Disc degeneration grade 5	0.73	0.87	0.79
Lumbar disc herniation (sagittal)	0.86	0.88	0.87
Lumbar disc herniation (axial)	0.92	0.91	0.91
High intensity zone (sagittal)	0.84	0.78	0.81
High intensity zone (axial)	0.74	0.77	0.75

## 4 Discussion

Pfirrmann grading, disc herniation, and HIZ are important MRI indicators to evaluate the intervertebral discs. Pfirrmann grading assesses the degree of disc degeneration based on its composition and morphology, providing valuable insights for predicting disease progression and guiding treatment strategies (Pfirrmann et al., 2001). Disc herniation is a common cause of LBP and radicular symptoms. While disc bulgings and herniations are frequently observed in imaging reports, not all cases are clinically symptomatic. Focus should primarily be on herniations causing significant nerve compression, which is the main concern of this study. HIZ is closely associated with annular tears and inflammatory responses, and is considered another important indicator of LBP (Wang and Hu, 2012). Furthermore, incomplete annular integrity may contribute to the progression of disc herniation.

Despite their clinical significance, interpreting MRI is intensive and time-consuming. Therefore, an auxiliary diagnostic model that can automatically identify the signs was developed in this study. Validation on both internal and external test sets demonstrated the model’s satisfactory capability in diagnosing and grading lumbar disc degeneration. Compared to prior models (Table 6), the proposed model offers several advantages. First, most existing models adopt a multi-stage approach, requiring manual or automated cropping of regions of interest. By integrating detection and classification tasks into a single-stage framework, the proposed model achieves efficient processing with reduced computational complexity. Second, this multi-task model achieved results comparable to those of single-task models. This design enhances its clinical applicability as a promising tool for assisting clinicians in routine practice.

**TABLE 6:** Performance comparison of the proposed model with previous studies.

	Precision	Recall
Disc herniation on axial images^a^		
Su et al. (2022)	0.80	0.71
Sustersic et al. (2022)	1.00	0.95
Zhang et al. (2023)	0.78	0.83
Ours	0.90	0.93
Grading of disc degeneration		
Natalia et al. (2024)	0.88	0.88
Gao et al. (2021)	0.86	0.86
Ours	0.88	0.88

^a^ According to the evaluation criteria for disc herniation used in this study, disc bulging or grade 1 herniation were classified as normal when calculating the diagnostic performance of previous models.

Several studies have attempted to automate the grading of disc degeneration. Since the development of SpineNet in 2017 (Jamaludin, Kadir, and Zisserman, 2017), researchers have proposed using various algorithmic models, including VGG-16 and Inception v3 (Soydan et al., 2023; Niemeyer et al., 2024). In this study, a modified YOLOv5 algorithm framework was employed, and the results demonstrated a relatively satisfactory classification performance. Notably, the primary issue with the automatic grading of disc degeneration may stem from the grading standard itself. Disc degeneration is a continuous process; however, the Pfirrmann grading system is a subjective, qualitative standard. Therefore, even clinical experts may struggle to definitively classify a disc, and the intraobserver coefficient can be low (Gao et al., 2021). To solve this problem, Gao et al. (2021) proposed adjusting the loss function to maximize the distance between the samples and the classification hyperplane. Although this theoretically improves classification performance, it also carries the risk of exaggerating the differences between the degrees of degeneration. We believe that minor deviations in grading typically do not directly affect clinical decision-making. In our test results, over 95% of image detections were evaluated as acceptable by a clinical expert. This result preliminarily meets the clinical requirements. An alternative strategy could be to introduce transitional grades (Niemeyer et al., 2021), which may more accurately reflect the natural progression of disc degeneration.

Given the high incidence of lumbar disc herniation, research on its automatic diagnosis has become a current hotspot. Over the past 2 years, researchers have achieved automatic grading and classification of disc herniation (Sustersic et al., 2022; Xu et al., 2024), resulting in the generation of more accurate and comprehensive imaging reports. However, these functions rely on extensive data annotation and training. Moreover, for multitask algorithms such as lumbar spine MRI interpretation, the inclusion of too many label types may increase the operating costs of the system, making its clinical deployment more challenging. For disc herniation, the imaging characteristics are often similar. However, no clear diagnostic imaging standards have been established (Li et al., 2015), leading to significant annotation noise. Therefore, in this study, a positive annotation strategy was adopted for training the automatic diagnostic model, which significantly reduced the number of labels and helped mitigate noise and overfitting. A previous study showed that this method can achieve performance comparable to that of fully labeled classifiers and offers certain advantages in multitask learning (Yuan et al., 2023). The proposed algorithm incorporates an SE-like attention mechanism and a residual calculation module, which enhance the detection of small targets such as disc herniation and HIZ. This approach further improves the performance of positive-label learning and assists clinicians in identifying potentially symptomatic disc herniations.

It is worth noting that the disc-wise diagnostic metrics for lumbar disc herniation demonstrated higher recall but lower precision compared to slice-wise metrics in both internal and external tests. This is because even if the model missed some lesions, a disc can still be diagnosed as positive due to the presence of typical lesions in other slices. For the same reason, false positives may also increase. This comparison highlights the importance of conducting a detailed analysis of individual slices from the original images. In clinical practice, accurate slice-wise diagnosis is essential, as it not only confirms whether a disc is pathological but also provides detailed information about the lesion’s location and boundaries. Such information can help clinicians correlate imaging abnormalities with clinical symptoms, assess disease severity, and develop treatment plans accordingly.

To the best of our knowledge, no previous studies have explored automatic detection methods for HIZ. The imaging manifestations of HIZ vary; it can appear in either the anterior or posterior portions of the disc and present in multiple forms, including rim, round, and fissure types. Our annotation strategy encompassed all HIZ types, which increased the detection complexity of the model. In addition, studies have indicated that HIZ is only likely to have clinical significance if it appears on at least two consecutive MRI slices, and in some patients, typical changes are observed on both T1W and T2W images (Wang and Hu, 2012; Shan et al., 2017). This indicates that a more clinically applicable diagnostic model needs to be of multi-input and multi-class. Therefore, a dedicated study on the automatic diagnosis of HIZ is currently being planned.

**FIGURE 7:**
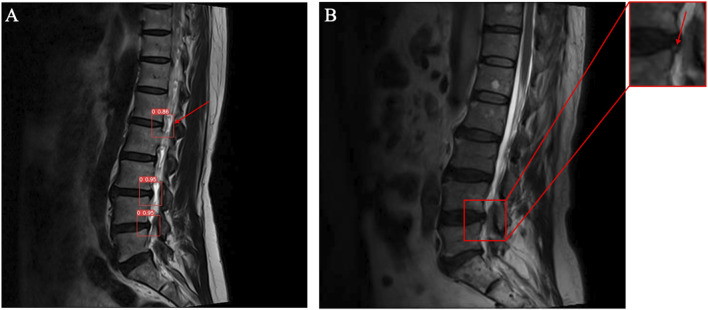
Illustrations of the incorrect diagnostic results of the model. **(A)** A mild disc bulge was misdiagnosed as disc herniation. **(B)** A small HIZ lesion was missed by the model.

Furthermore, the errors in the diagnostic and classification results of the deep-learning model were analyzed. For disc degeneration grading, the model demonstrated unstable performance in distinguishing grades 2–4. As previously discussed, this is mainly due to the inherently vague boundaries between these grades. Although the model exhibited classification capabilities similar to those of human clinicians in the test set, human clinicians nearly never make errors >1 grade. In future improvements, the loss function could be adjusted to solve this problem, for instance, by introducing grade smoothing or grade-weighted loss functions to tie the penalty more closely to the grading discrepancy and avoid significant misclassification. Regarding disc herniation and HIZ, Figure 7 highlights two typical detection errors. The model misdiagnosed a mild disc bulge as herniation and revealed insufficient detection capacity for smaller and marginally located HIZ. Balancing the sensitivity and specificity of detecting these two types of lesions may be a key future challenge. The model should be adapted according to the clinical application, prioritizing higher sensitivity for screening purposes or higher specificity for identifying symptomatic lesions. Moreover, for disc herniation detection, the model demonstrated similar performance on the external test set as it did on the internal test set. However, for the HIZ and disc degeneration grading, the results on the external test set showed a decline. This may be because the detection of the latter can be influenced by factors such as image contrast and resolution. This proposes that further training with multicenter data may be necessary in future studies.

The current algorithm still has some areas for improvement. We plan to conduct further studies on the following aspects: First, the current auxiliary diagnostic model does not incorporate automatic segmentation technology. In future work, automatic segmentation algorithms can be applied to predefined regions of interest, which would enhance the diagnostic performance and interpretability of the model. Second, the current model cannot medically localize the detected lesions. We plan to develop localization algorithms based on image segmentation technology (e.g., L4/5 disc herniation). Third, the integration of the existing image detection models with natural language processing (NLP) is feasible in the future (Bacco et al., 2022; Santomartino et al., 2024). For example, not all abnormal changes in MR images result in clinical symptoms. NLP can assist in localizing the responsible segment by analyzing the symptoms reported by the patients. Additionally, NLP can be used to integrate the output of image detection and generate radiological diagnostic reports. These techniques will help promote the clinical application of the automated diagnostic model.

This study has some limitations. The dataset used to train the model is still relatively limited; thus, supplementing data from different populations, pathologies, and scanners is necessary to minimize bias. Cross-validation was not performed during the training process, which may have potentially affected the model’s generalizability and robustness. Although the model’s diagnostic performance has undergone preliminary external validation, larger-scale independent validation is needed for further assessment. The proposed model is developed using supervised learning based on clinician-labeled data, which may limit its ability to accurately diagnose images where there is disagreement among human doctors. It is necessary to explore additional training methodologies to enhance its diagnostic performance in the context of complex diseases in the future. Moreover, in future research, new MRI techniques or precise and quantitative diagnostic criteria may be implemented.

## 5 Conclusion

The proposed deep learning model is based on a relatively lightweight multi-task framework and achieves satisfactory diagnostic and classification capabilities for lumbar disc degenerative diseases. Internal and external validations indicated that the model demonstrates diagnostic performance comparable to that of human clinicians. However, future research should focus on clinically driven model optimization. Key directions include training and testing the model on larger datasets to improve its generalizability and incorporating diverse input data to develop a multimodal diagnostic model suitable for clinical applications.

## Data Availability

The raw data supporting the conclusions of this article will be made available by the authors, without undue reservation.
